# Virtual Reality Body Exposure and Attentional Bias Modification in the Treatment of Adolescents With Anorexia Nervosa

**DOI:** 10.1002/cpp.70273

**Published:** 2026-04-14

**Authors:** Mariarca Ascione, Marta Carulla‐Roig, Franck‐Alexandre Meschberger‐Annweiler, Eduardo Serrano‐Troncoso, Anna Blasco‐Martínez, Fernando Guerrero‐Álvarez, Helena Miquel‐Nabau, María Teresa Mendoza‐Medialdea, Bruno Porras‐Garcia, Marta Ferrer‐Garcia, Manuel Moreno‐Sánchez, José Gutierrez‐Maldonado

**Affiliations:** ^1^ Department of Clinical Psychology and Psychobiology Universitat de Barcelona Barcelona Spain; ^2^ Institute of Neuroscience Universitat de Barcelona Barcelona Spain; ^3^ Department of Child and Adolescent Psychiatry and Psychology Hospital Sant Joan de Déu of Barcelona Barcelona Spain; ^4^ Department of Psychology Universidad de Jaén Jaén Spain; ^5^ Brain, Cognition and Behavior Research Group Consorci Sanitari de Terrassa Terrassa Spain; ^6^ Department of Basic Sciences Universitat Internacional de Catalunya Sant Cugat del Vallès Spain; ^7^ Department of Cognition, Development and Educational Psychology University of Barcelona Barcelona Spain

**Keywords:** adolescents, anorexia nervosa, attentional bias, eye‐tracking, mirror exposure therapy, virtual reality

## Abstract

Anorexia nervosa (AN) is a severe psychiatric disorder characterized by intense fear of gaining weight and persistent body image disturbance. Virtual reality–based mirror exposure therapy (VR‐MET) may reduce body‐related fear through embodied exposure, while attentional bias modification training (ABMT) may enhance exposure learning by promoting balanced attentional allocation. This controlled clinical study tested the hypothesis that adjunctive VR‐MET would improve clinical outcomes beyond treatment as usual (TAU) alone, and that preceding VR‐MET with ABMT would produce additional benefits in adolescent females with AN. Seventy‐five female adolescents with AN were allocated to TAU, TAU+VR‐MET or TAU+ABMT+VR‐MET. Assessments were conducted pre‐ and post‐intervention. Outcomes included eye‐tracking indices of attentional bias (number of fixations, complete fixation time), state anxiety and fear of gaining weight, BMI and eating disorder–related measures. Compared with TAU alone, both VR‐based conditions showed greater reductions in state anxiety and fear of gaining weight. State body dissatisfaction decreased significantly only in the TAU+VR‐MET group. No significant changes were observed for BMI or most trait‐level eating disorder measures. ABMT did not enhance clinical outcomes beyond VR‐MET. Within the short‐term assessment window, adjunctive VR‐MET was associated with reductions in state‐dependent emotional responses in adolescents with AN. Effects on trait‐level symptoms were limited, and ABMT did not confer additional benefit in this unselected sample. Fully randomized studies with larger samples, extended exposure protocols, and follow‐up assessments are needed to determine durability and broader clinical impact.

## Summary

- Adjunctive virtual reality–based mirror exposure therapy may reduce short‐term state anxiety and fear of gaining weight in adolescent females with anorexia nervosa when added to treatment as usual.
- Short‐term virtual reality–based mirror exposure therapy primarily impacts state‐dependent emotional responses, whereas trait‐level eating disorder symptoms (e.g., drive for thinness and trait body dissatisfaction) may require longer or more intensive interventions.
- Adding attentional bias modification training before virtual reality–based mirror exposure therapy did not enhance clinical outcomes in an unselected clinical sample, suggesting that attentional retraining may be more beneficial when targeted to patients with pronounced baseline attentional bias.

## 1 Introduction

Anorexia nervosa is a severe psychiatric disorder characterized by significantly low body weight, intense fear of gaining weight and persistent body image disturbance (American Psychiatric Association 2013). It is associated with high psychiatric comorbidity and one of the highest mortality rates among mental disorders (American Psychiatric Association 2013). Although onset typically occurs during adolescence, over 30% of individuals develop a chronic course persisting into adulthood (Smink et al. 2012). Standard treatments focus primarily on weight restoration and medical stabilization, yet progress is often hindered by an enduring fear of gaining weight and body image disturbances.

Exposure‐based interventions aim to reduce negative emotional and evaluative responses to feared body‐related stimuli through repeated confrontation (Vocks et al. 2007; Delinsky and Wilson 2006). Mirror exposure therapy (MET) promotes emotional processing via sustained engagement with one's own body, facilitating habituation and new learning. Virtual reality–based mirror exposure therapy (VR‐MET) extends this approach by enabling interaction with a virtual body representation in an immersive, controlled environment (Delinsky and Wilson 2006; Perpiñá et al. 2003). By manipulating avatar body size while preserving the illusion of full‐body ownership, VR‐MET allows embodied simulation of weight‐related changes that are difficult to achieve in vivo and offers greater experimental control than imagery‐based exposure (Riva et al. 2021). Preliminary findings indicate that VR‐MET can reduce fear of gaining weight and body‐related distress in anorexia nervosa (Porras‐Garcia et al. 2021).

From an inhibitory learning perspective (Craske et al. 2014), exposure is most effective when feared stimuli are activated, and new, non‐threatening associations are encoded and retrieved across contexts. Attentional processes may critically shape this learning (Ferrer‐Garcia et al. 2021), as visual attention determines which body‐related cues are processed and integrated into threat representations. Attentional bias (AB), the preferential allocation of attention toward disorder‐relevant stimuli, is consistently observed in anorexia nervosa (Williamson et al. 2004), with disproportionate focus on negatively evaluated, weight‐related body areas (e.g., abdomen, hips and thighs) (Jansen et al. 2005; Kerr‐Gaffney et al. 2019; Rodgers and DuBois 2016; Roefs et al. 2008; Tuschen‐Caffier et al. 2015). Such patterns may reinforce negative self‐evaluation and emotional distress, contributing to disorder maintenance (Lee and Shafran 2004). Moreover, evidence suggests a bidirectional relationship between AB and body dissatisfaction (Rodgers and DuBois 2016; Smith and Rieger 2006; Stice et al. 2010). During exposure, rigid fixation on negatively evaluated regions may constrain perceptual processing and amplify distress, whereas more distributed attention may facilitate integrative body processing. Modifying maladaptive attentional patterns before exposure may therefore promote broader information sampling, reduce overprocessing of negatively evaluated regions and enhance corrective learning.

Attentional bias modification training (ABMT) directly targets attentional allocation by guiding gaze toward predefined stimulus locations. Initially developed for anxiety disorders (Hallion and Ruscio 2011), ABMT has been adapted for eating disorders and shows preliminary efficacy in reducing body‐related AB in non‐clinical samples (Meschberger‐Annweiler et al. 2023; Miquel‐Nabau et al. 2023) and in anorexia nervosa, with some evidence of concurrent reductions in body dissatisfaction (Ascione et al. 2023). Advances in VR and eye‐tracking enable ABMT delivery within immersive avatar‐based environments, allowing precise monitoring and manipulation of gaze distribution (Lutz et al. 2017). In the present study, ABMT was designed to promote balanced attention across weight‐related and non–weight‐related body areas before VR‐MET, with the aim of facilitating more integrative perceptual processing during exposure.

To date, no controlled clinical study has examined whether modifying attentional allocation before immersive body exposure enhances emotional or clinical outcomes in adolescents with anorexia nervosa.

This controlled clinical study investigated the effectiveness of VR‐MET, delivered alone or preceded by ABMT, as an adjunct to treatment as usual (TAU) in adolescent females with anorexia nervosa. Participants were allocated to: (1) TAU, (2) TAU+VR‐MET or (3) TAU+ABMT+VR‐MET. It was hypothesized that adjunctive VR‐MET would reduce anxiety, fear of gaining weight and body image–related outcomes relative to TAU alone. It was further hypothesized that preceding VR‐MET with ABMT would yield incremental benefits by promoting more balanced attentional allocation during exposure.

## 2 Methods

### 2.1 Clinical Sample

Seventy‐five adolescent females diagnosed with anorexia nervosa were recruited from the Eating Disorders Unit of Hospital Sant Joan de Déu in Barcelona. Sample size estimation was informed by effect sizes reported in a controlled study investigating body exposure with sustained attentional focus on specific body areas (Jansen et al. 2016). Although this study differed in design and population (i.e., non‐clinical samples), it provided the closest available reference for an a priori power estimation.

Participants were assigned to one of three study conditions. Due to recruitment constraints and delays in ABMT software implementation, a subset of the TAU and TAU + VR‐MET samples was derived from a previously completed randomized controlled trial conducted by the same research group (Porras‐Garcia et al. 2021), which employed identical TAU and VR‐MET protocols and was conducted within the same clinical unit. Only participants meeting the current study's inclusion and exclusion criteria were retained to ensure comparability. Consequently, the study employed a partially randomized design: newly recruited participants were randomly assigned to TAU, TAU + VR‐MET or TAU + ABMT + VR‐MET using block randomization with a computer‐generated sequence, whereas 21 historical participants were included only in the TAU (*n* = 12) and TAU + VR‐MET (*n* = 9) conditions.

**FIGURE 1:**
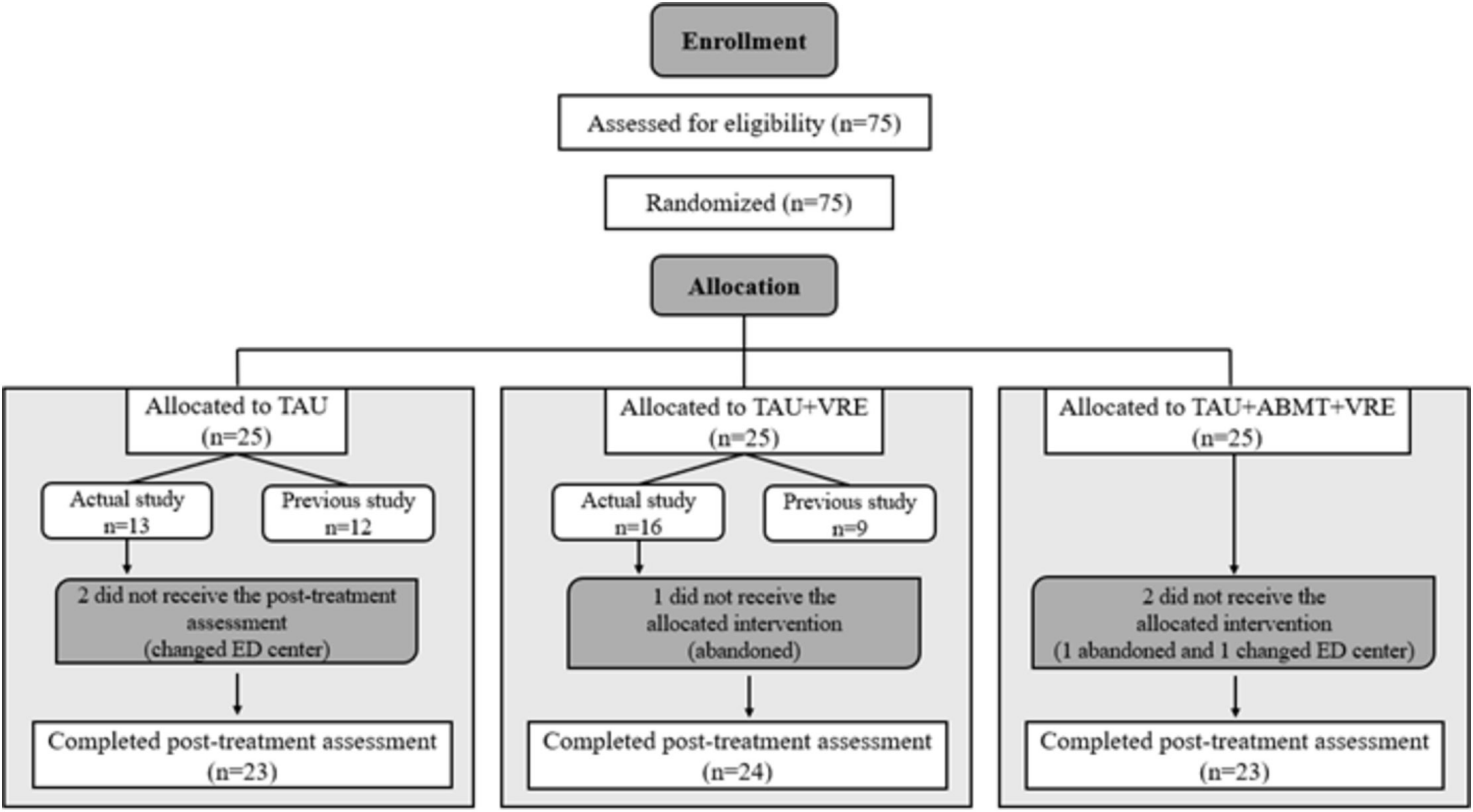
Participant flow throughout the study.

Five participants discontinued participation before the post‐intervention assessment (2 in TAU, 1 in TAU + VR‐MET and 2 in TAU + ABMT + VR‐MET). Attrition was due to reasons not directly related to the intervention: three participants were transferred to other clinical centres, and two withdrew for logistical reasons. The final sample consisted of 70 participants who completed all assessments (see Figure 1).

Inclusion criteria were: (a) female adolescents aged 12–17 years; (b) a DSM‐5 diagnosis of anorexia nervosa (American Psychiatric Association 2013); and (c) body weight below the expected range for age, height and sex. Diagnoses were established by specialized clinicians based on comprehensive psychological, nutritional and medical evaluations, including structured clinical interviews.

The sample was restricted to females to reduce heterogeneity and increase internal validity, given known sex differences in prevalence and clinical presentation (Van Eeden et al. 2021; Porras García et al. 2019; Riva et al. 2023). The selected age range reflects the typical onset period of anorexia nervosa (Van Eeden et al. 2021) and corresponds to a developmental stage in which body image and attentional processes are particularly salient (Hebebrand et al. 2024).

Exclusion criteria included severe psychiatric comorbidities, significant cognitive impairment, visual or auditory deficits interfering with VR‐MET, epilepsy, pregnancy and cardiac arrhythmia.

### 2.2 Measures

All measures were administered at pre‐ and post‐intervention.

#### 2.2.1 Attentional Biases

AB toward specific body regions was assessed using eye‐tracking while participants viewed their avatar in a virtual mirror. Fixations were defined as gaze maintained for 100–200 ms (Jacob and Karn 2003). Based on the framework of the Physical Appearance State and Trait Anxiety Scale (PASTAS) (Reed et al. 1991), two categories of areas of interest (AOIs) were defined: weight‐related AOIs (stomach, hips, waist, thighs and legs) and non–weight‐related AOIs (all remaining body regions). Weight‐related AB reflects attention directed toward body areas typically associated with weight and shape concerns, whereas non–weight‐related AB reflects attention allocated to non‐salient body regions. Consistent with prior eye‐tracking studies in anorexia nervosa (Kerr‐Gaffney et al. 2019), two indices were extracted for each AOI category: number of fixations (NF) and complete fixation time (CFT).

#### 2.2.2 Visual Analog Scale (VAS)

During VR exposure, participants rated state general anxiety, fear of gaining weight and the full‐body ownership illusion on 0–100 VASs (0 = not at all; 100 = completely).

#### 2.2.3 BMI

Weight and height were measured using a digital scale with an integrated stadiometer. Participants were assessed in underwear and without shoes. BMI was calculated as weight (kg)/height squared (m^2^).

#### 2.2.4 Eating Disorder Psychopathology (Trait): Body Dissatisfaction, Drive for Thinness and Fear of Gaining Weight

Trait eating disorder symptoms were assessed using the Spanish version (Elosua et al. 2010) of the Eating Disorder Inventory‐3 (EDI‐3) (Garner 2004). The Body Dissatisfaction (EDI‐BD; *α* = 0.78) and Drive for Thinness (EDI‐DT; *α* = 0.90) subscales were administered. The EDI‐BD evaluates dissatisfaction with body shape and weight, whereas the EDI‐DT assesses concerns related to dieting, weight and the pursuit of thinness. The item ‘I am terrified about weight gain’ was analysed separately as an index of trait fear of gaining weight. Higher scores indicate greater symptom severity.

#### 2.2.5 Body Anxiety (State)

State anxiety toward weight‐related body areas was assessed using the Weight subscale (8 items; *α* = 0.81) of the PASTAS (Reed et al. 1991), which captures cognitive, emotional and physiological components of appearance‐related anxiety. Higher scores indicate greater anxiety.

#### 2.2.6 State Body Dissatisfaction and Body Distortion

The Body Image Assessment Scale–Body Dimensions (BIAS‐BD) (Gardner et al. 2009) was used to assess state body dissatisfaction and body size distortion. Participants selected perceived and ideal body silhouettes from 17 standardized figures varying in body size, which were matched to each participant's BMI by the research team.

#### 2.2.7 Body Appreciation

Positive attitudes toward one's body were assessed using the Spanish version (Lobera and Ríos 2011) of the Body Appreciation Scale (BAS) (Avalos et al. 2005), which evaluates acceptance of physical features and rejection of unrealistic body ideals. Internal consistency in this sample was high (*α* = 0.91). Higher scores indicate greater body appreciation.

### 2.3 Instruments

#### 2.3.1 Hardware

Participants were immersed in a VR scenario using an HTC VIVE Pro Eye head‐mounted display equipped with dual OLED displays (2880 × 1600 resolution; 615 PPI). Movement tracking relied on five wireless trackers (head‐mounted display, two controllers and a pair of foot‐mounted trackers) synchronized with four SteamVR Base Station 2.0 units, enabling accurate full‐body tracking within a 10 × 10 m area. The head‐mounted display's integrated Tobii eye‐tracking system provided binocular gaze data at 120 Hz with a spatial accuracy of 0.5°–1.1°. Eye‐tracking calibration followed a standard 5‐point procedure.

#### 2.3.2 Software

The VR environment, developed in Unity 3D (version 5.6.1), consisted of a minimally furnished room with a large mirror positioned 1.54 m in front of the participant and two additional floor‐level reference boxes. An avatar, presented as the participant's reflection, reproduced their movements in real time, allowing both full‐body and first‐person perspectives. Avatars were created in Blender (version 2.78) and customized to match participants' height, silhouette and relevant physical features (e.g., skin tone and general appearance). To minimize visual discrepancies between participants and their avatars, they were displayed wearing black shoes, a grey cap and a head‐mounted display, which helped to mask individual facial characteristics and standardize appearance, thereby enhancing the plausibility of the virtual body representation.

### 2.4 Procedure

The study was approved by the Ethics Committees of the University of Barcelona (IRB00003099) and Hospital Sant Joan de Déu (PS‐21‐20) and was prospectively registered at ClinicalTrials.gov (NCT04786951). The protocol and reporting followed the TREND guidelines for non‐randomized studies (Des Jarlais et al. 2004). Participants were recruited from the eating disorders unit and received TAU, with adjunctive interventions depending on group allocation. TAU consisted of one of three clinical programs tailored to individual clinical needs and risk levels. The 11‐h day‐patient programme followed a cognitive‐behavioural therapy model and included nutritional rehabilitation, behavioural strategies to promote weight gain, individual and group psychotherapy and parental support within a structured daily schedule. The 6‐h day‐patient programme comprised the same therapeutic components but was delivered within a reduced schedule for patients transitioning from the 11‐h programme and presenting with lower clinical risk. The home‐treatment programme served as transitional care between hospitalization and outpatient treatment and focused on medical stabilization, weight restoration, family involvement, psychoeducation, emotional regulation and social skills development. Evidence‐based treatments, including cognitive‐behavioural therapy and family‐based therapy, were delivered either in person or remotely.

Participants were randomly assigned to one of three conditions:

1. TAU group: Received the standard eating disorders unit protocol.
2. TAU + VR‐MET group: Received the standard protocol plus five weekly VR‐MET sessions.
3. TAU + ABMT + VR‐MET group: Received the standard protocol plus five weekly VR‐MET sessions, each preceded by ABMT.

#### 2.4.1 Assessment Sessions

Pre‐ and post‐intervention assessments were conducted immediately before and after the five‐week intervention period and lasted approximately 1 h. Participants and their parents or legal guardians provided informed consent, and eligibility was confirmed by clinical staff. Participants were assigned identification codes to ensure anonymity.

To personalize the virtual avatar, a standardized front‐facing photograph was taken and used to adjust the avatar's body silhouette to approximate the participant's body shape. While one researcher adjusted the avatar's body characteristics, another administered the assessment measures. Once the avatar had been personalized, a full‐body ownership illusion was induced through visuomotor and visuotactile stimulation. During visuomotor stimulation, participants' real and virtual movements were aligned using head, hand and foot tracking, allowing both first‐person and mirrored perspectives. Visuotactile stimulation was delivered by a gender‐matched researcher using one of the HTC Vive handheld controllers to apply synchronous touches to corresponding real and virtual body regions. Additional details are reported in (Porras‐Garcia et al. 2020).

Following illusion induction, participants completed a VAS assessing state anxiety, fear of gaining weight and body ownership. Gaze behaviour was then recorded using the integrated eye tracker while participants viewed their avatar in a virtual mirror for 30 s (Jansen et al. 2005; Roefs et al. 2008). To reduce expectancy effects, participants were informed that the task was intended for body‐position calibration and were instructed to remain still. The head‐mounted display was removed after task completion.

#### 2.4.2 Virtual Reality Body Exposure Sessions (TAU + VR‐MET Group)

Participants in the TAU + VR‐MET group received five weekly sessions lasting approximately 45 min each. After induction of the body ownership illusion, participants were exposed to an avatar representing their current BMI. Exposure followed a hierarchical structure, with attention directed sequentially across body regions from head to feet, consistent with pure MET protocols (Moreno‐Domínguez et al. 2012). Participants were encouraged to verbalize thoughts and emotions associated with each body region, and anxiety levels were assessed every 2 min using VAS. Compared with alternative approaches (e.g., non‐judgemental or cognitive dissonance–based exposure), pure mirror exposure has demonstrated superior efficacy in reducing body dissatisfaction (Moreno‐Domínguez et al. 2012; Hilbert et al. 2002; Luethcke et al. 2011).

Across sessions, the avatar's body size was progressively increased to simulate weight gain toward an individualized healthy BMI, determined based on WHO growth charts (World Health Organization [WHO] 2004) and premorbid weight. Progression required at least a 40% reduction in anxiety relative to the previous session; otherwise, the same exposure level was repeated to facilitate within‐session habituation. Participants were informed about the rationale of exposure but were not explicitly informed of BMI changes.

Each session concluded with immersion in relaxing virtual environments (e.g., forest and garden), followed by a therapist‐led debriefing. Additional support was provided if anxiety levels remained elevated. All sessions were conducted by a licensed psychologist with expertise in eating disorders.

#### 2.4.3 AB Modification Training Sessions (TAU + ABMT + VR‐MET Group)

Before each VR‐MET session, participants in the TAU + ABMT + VR‐MET group completed an ABMT protocol adapted from (Smeets et al. 2011) to reduce AB toward body areas and promote balanced attentional distribution. This protocol has demonstrated efficacy in modifying AB in both healthy individuals (Meschberger‐Annweiler et al. 2023; Miquel‐Nabau et al. 2023) and patients with anorexia nervosa (Ascione et al. 2023). Participants were informed that the objective of the task was to encourage a more balanced distribution of attention across different body regions; however, the specific attentional training purpose of the task was not disclosed.

**FIGURE 2:**
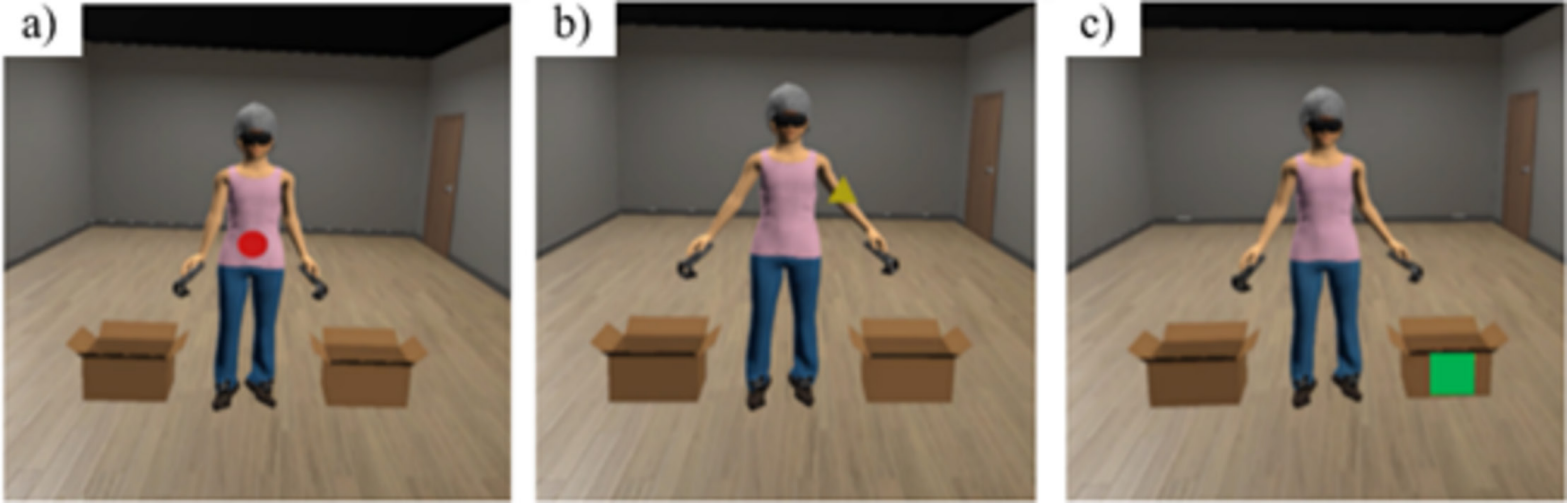
Attentional bias modification training visual representation: geometric figures appearing on a weight‐related body part (a), on a non‐weight‐related body part (b) and on a neutral stimulus (c).

Within a virtual mirror environment, participants observed their avatar while geometric shapes of varying colours and forms appeared on specific body regions. Participants were instructed to fixate on each shape for 4 s; if gaze deviated from the target, the task paused until fixation was re‐established, ensuring controlled attentional allocation. Task demands alternated between identifying shape and colour to maintain engagement. Stimuli were presented with equal probability on weight‐related and non–weight‐related body areas (45% each), with the remaining 10% presented adjacent to the body (see Figure 2). Each session consisted of 150 trials divided into two blocks lasting approximately 10–15 min, consistent with prior recommendations for ABMT duration (Meschberger‐Annweiler et al. 2023).

### 2.5 Statistical Analysis

Eye‐tracking data were processed using Open Gaze and Mouse Analyser (OGAMA), which converted raw gaze recordings into analysable metrics. AB indices were calculated by subtracting fixations on non–weight‐related areas from weight‐related areas (e.g., 15 W‐AOIs −5 NW‐AOIs = 10). Positive values reflected bias toward weight‐related areas, negative values toward non–weight‐related areas and values near zero indicated balanced attention. Indices were computed separately for NF and CFT.

All analyses were conducted using IBM SPSS Statistics (Version 29). Effect sizes were reported as partial eta squared (η^2^_p_), with conventional thresholds of 0.01 (small), 0.06 (medium) and 0.14 (large) (Cohen 1988). Baseline group differences were examined using one‐way ANOVAs for continuous variables and chi‐square tests for categorical variables. Because a subset of participants was drawn from a previous study, cohort comparisons (historical vs. newly recruited participants) were performed using independent‐samples *t*‐tests and chi‐square tests. Variables showing significant baseline differences were entered as covariates in subsequent analyses; categorical covariates with more than two levels were dummy coded.

Mixed‐design analyses of covariance (ANCOVAs) were conducted to examine group (TAU, TAU+VR‐MET, TAU+ABMT+VR‐MET) × time (pre vs. post) effects on BMI, eating disorder–related measures, AB indices and VAS ratings, adjusting for relevant covariates.

Assumptions of normality and homogeneity of variance were evaluated before analysis. Although some violations were observed (*p* < 0.05), mixed‐design ANOVA models are considered robust to moderate deviations from normality in samples with comparable group sizes (Schmider et al. 2010). Analyses were therefore conducted as planned, with cautious interpretation.

Attrition was low (6.7%) and unrelated to intervention allocation. Both intention‐to‐treat (ITT) and per‐protocol (PP) analyses were performed. Missing post‐intervention data were handled using last observation carried forward under the assumption of missing completely at random.

## 3 Results

### 3.1 Descriptive Results

Participants had a mean age of 15.16 years (SD = 1.44; range = 12–17) and a mean BMI of 16.95 kg/m^2^ (SD = 1.30; range = 12.91–19.60). The mean BMI z‐score was −1.41 (SD = 0.81; range = −4.63 to −0.40), corresponding to a mean BMI percentile of 13.08 (SD = 9.93; range = 0.10–34.09). Additional descriptive characteristics are presented in Table 1.

**TABLE 1:** Baseline demographic and clinical characteristics of the study groups.

Variable	Control Group 1 TAU (*n* = 25)	Control Group 2 TAU + VR‐MET (*n* = 25)	Experimental group TAU + ABMT + VR‐MET (*n* = 25)	Statistic	*p*
Age and BMI, M (SD)					
Age, years	15.32 (1.18)	14.76 (1.50)	15.40 (1.58)	*F*(2,72) = 1.47	0.235
BMI, kg/m^2^	17.30 (0.97)	16.87 (1.10)	16.68 (1.70)	*F*(2,72) = 1.49	0.232
BMI percentile	14.94 (10.47)	13.55 (9.14)	10.74 (10.08)	*F*(2,72) = 1.16	0.318
BMI z‐score	−1.21 (0.64)	−1.29 (0.69)	−1.64 (1.00)	*F*(2,72) = 2.06	0.135
Main diagnosis, *n* (%)				*χ* ^*2*^(2) = 2.08	0.353
AN‐R type	23 (92)	25 (100)	24 (96)		
AN‐P type	2 (8)	0 (0)	1 (4)		
Comorbid diagnosis, *n*				N/A	N/A
GAD	0	2	0		
MDD	2	0	5		
PDD	2	1	3		
AvPD	0	0	1		
PTSD	0	0	2		
OCD	1	2	0		
SAD	0	0	2		
UPD	1	0	1		
UAD	1	2	0		
UDD	2	0	0		
BDP	1	0	0		
Number of comorbid diagnoses, *n* (%)				*χ* ^*2*^(4) = 3.27	0.513
0	16 (64)	19 (76)	13 (52)		
1	8 (32)	5 (20)	10 (40)		
2	1 (4)	1 (4)	2 (8)		
Any psychotropic medication^a^, *n* (%)				*χ* ^*2*^(2) = 4.72	0.095
Yes	17 (68)	18 (72)	23 (92)		
No	8 (32)	7 (28)	2 (8)		
Medication type, *n* (%)				*χ* ^*2*^(14) = 18.63	0.180
None	8 (32)	7 (28)	2 (8)		
Antidepressant	4 (16)	3 (12)	5 (20)		
Antipsychotic	0 (0)	0 (0)	1 (4)		
Anxiolytic	0 (0)	5 (20)	2 (8)		
Antidepressant/antipsychotic	5 (20)	2 (8)	3 (12)		
Antidepressant/anxiolytic	1 (4)	4 (16)	6 (24)		
Antipsychotic/anxiolytic	1 (4)	1 (4)	0 (0)		
Antidepressant/antipsychotic/anxiolytic	6 (24)	3 (12)	6 (24)		
Treatment program, *n* (%)				*χ* ^*2*^(4) = 13.08	0.011
Day‐patient (11 h/day)	18 (72)	15 (60)	10 (40)		
Day‐patient (6 h/day)	6 (24)	1 (4)	5 (20)		
Home‐treatment	1 (4)	9 (36)	10 (40)		
Eating disorder psychopathology (EDI), M (SD)					
Body dissatisfaction	29.24 (5.67)	24.76 (6.11)	29.00 (6.42)	*F*(2,72) = 4.30	0.017
Drive for thinness	22.68 (5.42)	22.12 (5.76)	22.32 (7.20)	*F*(2,72) = 0.53	0.949
Fear of gaining weight	3.60 (1.16)	3.56 (1.00)	3.52 (1.12)	*F*(2,72) = 0.33	0.967

*Note:* Values are means (M) and standard deviations (SD) for continuous variables and counts and percentages (*n* [%]) for categorical variables.

Abbreviations: AN‐P, anorexia nervosa purgative subtype; AN‐R, anorexia nervosa restrictive subtype; AvPD, avoidant personality disorder; BMI, body mass index; BPD, borderline personality disorder; EDI, Eating Disorder Inventory; GAD, generalised anxiety disorder; MDD, major depressive disorder; N/A, not applicable; OCD, obsessive‐compulsive disorder; PDD, persistent depressive disorder; PTSD, post‐traumatic stress disorder; SAD, social anxiety disorder; UAD, unspecified anxiety disorder; UDD, unspecified depressive disorder; UPD, unspecified personality disorder.

^a^ Any psychotropic medication indicates treatment with at least one psychotropic drug at baseline.

**FIGURE 3:**
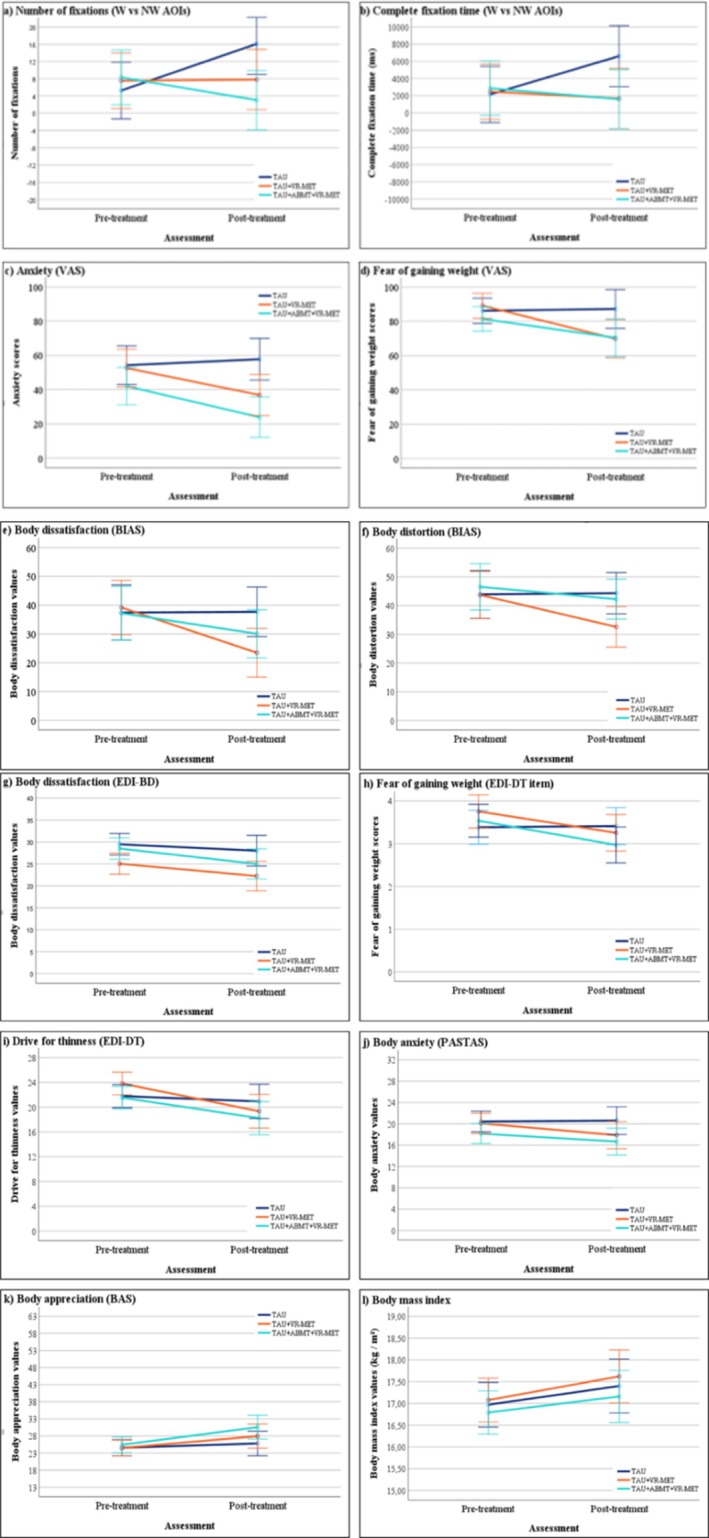
(a, b). Means of the experimental and control groups in the two assessment conditions (pre‐treatment and post‐treatment) in the number of fixations (a) and complete fixation time (b). (c, d). Means of the experimental and control groups in the two assessment conditions (pre‐treatment and post‐treatment) in anxiety—VAS (c) and fear of gaining weight—VAS (d). (e–l). Means of the experimental and control groups in the two assessment conditions (pre‐treatment and post‐treatment) in body distortion (e), body dissatisfaction—BIAS (f), body dissatisfaction—EDI‐BD (g), fear of gaining weight—EDI‐DT (h), drive for thinness (i), body anxiety (j), body appreciation (k), body mass index (l).

Baseline differences among the three experimental groups were examined using one‐way ANOVAs for continuous variables and chi‐square tests for categorical variables. No significant baseline differences were observed for age, BMI, BMI z‐scores, BMI percentiles, primary diagnosis, number of comorbidities or psychotropic medication use (all *p*s > 0.05). A significant difference emerged in the distribution of treatment programs across groups, *χ* ^2^(4) = 13.08, *p* = 0.011. In addition, a significant group difference was observed for body dissatisfaction (EDI‐BD), *F*(2, 72) = 4.30, *p* = 0.017. Post hoc comparisons indicated that the TAU + VR‐MET group reported lower body dissatisfaction than both the TAU group (MD = −4.48, SE = 1.71, *p* = 0.029) and the TAU + ABMT + VR‐MET group (MD = −4.24, SE = 1.71, *p* = 0.042) (see Table 1). To evaluate potential cohort effects, exploratory comparisons were conducted between historical and newly recruited participants, revealing significant differences in BMI, *t*(73) = −2.74, *p* = 0.008, psychotropic medication use, *χ* ^2^(1) = 19.78, *p* < 0.001 and treatment programme allocation, *χ* ^2^(2) = 21.71, *p* < 0.001 (see Table S1). Based on these results, baseline body dissatisfaction (EDI‐BD), treatment program, BMI and psychotropic medication use were included as covariates in subsequent mixed‐design ANCOVAs to adjust for baseline and cohort‐related differences. Means and standard errors for all outcomes are presented in Figure 3.

### 3.2 Body‐Related AB

Two‐way mixed ANCOVAs were conducted to examine the effects of group (TAU, TAU + VR‐MET, TAU + ABMT + VR‐MET) and time (pre‐ vs. post‐treatment) on CFT and NF, controlling for baseline body dissatisfaction (EDI‐BD), BMI, treatment programme and psychotropic medication use.

For NF, a significant group × time interaction emerged, *F*(2, 67) = 4.63, *p* = 0.013, η_p_^2^ = 0.121. Pairwise comparisons indicated a significant increase in NF from pre‐ to post‐treatment only in the TAU group (MD = −10.84, SE = 3.73, *p* = 0.005). No significant changes were observed in the VR‐based conditions. At post‐treatment, the TAU group showed significantly higher NF compared with the TAU + ABMT + VR‐MET group (MD = 13.06, SE = 5.09, *p* = 0.038). PP analyses replicated this pattern.

For CFT, the group × time interaction was not statistically significant, *F*(2, 67) = 2.61, *p* = 0.081, *η_p_* ^2^ = 0.072, and no significant main effects were observed (*p*s > 0.05).

At baseline, the mean CFT bias index was 2507.41 ms (SD = 7990.58; maximum possible viewing time = 30,000 ms). The mean NF bias index (weight‐related minus non‐weight‐related fixations) was 7.03 (SD = 15.91). For reference, during the 30‐s viewing period, the total NF would typically fall within a theoretical range of approximately 150–300.

### 3.3 State General Anxiety and Fear of Gaining Weight (VASs)

Two‐way mixed‐design ANCOVAs were conducted to examine the effects of group and time on state anxiety and state fear of gaining weight VASs, controlling for baseline body dissatisfaction (EDI‐BD), BMI, treatment programme and psychotropic medication use.

For anxiety, a significant group × time interaction was observed, *F*(2, 67) = 3.32, *p* = 0.042, *η_p_* ^2^ = 0.090, as well as a significant main effect of group, *F*(2, 67) = 5.29, *p* = 0.007, *η_p_* ^2^ = 0.136. Significant reductions in anxiety were observed from pre‐ to post‐treatment in both the TAU + VR‐MET group (MD = 15.73, SE = 6.20, *p* = 0.014) and the TAU + ABMT + VR‐MET group (MD = 18.10, SE = 6.11, *p* = 0.004). At post‐treatment, the TAU group reported significantly higher anxiety than the TAU + ABMT + VR‐MET group (MD = 33.83, SE = 8.73, *p* < 0.001); however, the difference between the TAU and TAU + VR‐MET groups did not reach statistical significance, with numerically higher anxiety in the TAU group (MD = 20.89, SE = 8.90, *p* = 0.066). PP analyses yielded comparable results for the main effect of group, with the TAU group reporting significantly higher anxiety than the TAU + ABMT + VR‐MET group; however, the group × time interaction did not reach statistical significance, *F*(2, 62) = 2.90, *p* = 0.062, *η_p_* ^2^ = 0.086.

For fear of gaining weight, a significant group × time interaction was observed, *F*(2, 67) = 3.99, *p* = 0.023, *η_p_* ^2^ = 0.107. Fear of gaining weight significantly decreased from pre‐ to post‐treatment in both the TAU + VR‐MET group (MD = 19.29, SE = 4.87, *p* < 0.001) and the TAU + ABMT + VR‐MET group (MD = 10.95, SE = 4.80, *p* = 0.026). PP analyses replicated these findings.

### 3.4 Eating Disorders Measures

Two‐way mixed ANCOVAs were conducted to examine the effects of group and time on eating disorder–related outcomes, controlling for baseline body dissatisfaction (EDI‐BD; except when EDI‐BD was the outcome), BMI (except when BMI was the outcome), treatment programme and psychotropic medication use.

A significant group × time interaction was observed for state body dissatisfaction (BIAS‐BD), *F*(2, 67) = 3.98, *p* = 0.023, *η_p_* ^2^ = 0.106. Significant reductions were observed in the TAU + VR‐MET group (MD = 15.70, SE = 3.82, *p* < 0.001). The reduction observed in the TAU + ABMT + VR‐MET group did not reach statistical significance (MD = 7.15, SE = 3.77, *p* = 0.062). PP analyses yielded similar results.

For body distortion (BIAS‐BD), the group × time interaction did not reach statistical significance, *F*(2, 67) = 3.10, *p* = 0.052, *η_p_* ^2^ = 0.085, whereas a significant main effect of time was observed, *F*(1, 67) = 4.50, *p* = 0.038, *η_p_* ^2^ = 0.063. Scores decreased across groups from pre‐ to post‐treatment (MD = 5.00, SE = 1.72, *p* = 0.005). PP analyses replicated this effect.

For trait body dissatisfaction (EDI‐BD), a significant main effect of group was found, *F*(2, 68) = 3.57, *p* = 0.034, *η_p_* ^2^ = 0.095. Post hoc comparisons indicated that the TAU group reported significantly higher scores than the TAU + VR‐MET group (MD = 5.09, SE = 1.93, *p* = 0.032). PP analyses replicated these results.

No significant main effects or interactions were observed for BMI, body anxiety, body appreciation, drive for thinness or trait fear of gaining weight (EDI; *p*s > 0.05) in either the ITT or PP analyses.

## 4 Discussion

This controlled clinical study examined the effectiveness of VR‐MET, delivered alone or preceded by ABMT, as an adjunct to TAU in adolescent females with anorexia nervosa. Findings partially support the potential clinical utility of VR‐based interventions, showing reductions in state‐dependent emotional responses but no significant effects on more stable, trait‐like eating disorder features. No evidence emerged for the incremental benefit of ABMT when administered before VR‐MET in this unselected sample, as participants were not selected based on baseline AB.

Consistent with hypotheses, participants receiving VR‐MET (with or without ABMT) showed greater reductions in state anxiety and fear of gaining weight relative to TAU alone. These results align with findings from immersive body‐exposure research (Porras‐Garcia et al. 2021) and are consistent with exposure‐based models. Repeated confrontation with feared body‐related stimuli may reduce fear and anxiety through processes such as habituation or inhibitory learning (Craske et al. 2014). In the present study, repeated embodied exposure to progressively weight‐increased avatars may have allowed participants to experience feared body‐related changes in a controlled environment, facilitating a gradual reduction in anxiety responses. The induction of full‐body ownership through multisensory synchrony, consistent with prior VR research in anorexia nervosa (Porras‐Garcia et al. 2021), may have enhanced self‐referential processing and affective salience, thereby facilitating exposure‐based learning processes (Tsakiris 2010).

State body dissatisfaction decreased significantly only in the TAU+VR‐MET group. While MET is known to influence affective–evaluative components of body image (Porras‐Garcia et al. 2021; Key et al. 2002; Jansen et al. 2008; Vocks et al. 2008), the absence of improvement in the ABMT+VR‐MET group was unexpected. One speculative explanation is that externally guided attentional orientation immediately before exposure may have constrained spontaneous perceptual and emotional engagement during VR‐MET, although this mechanism was not directly assessed. Within inhibitory learning frameworks, corrective learning is optimized when individuals freely engage feared stimuli and generate expectancy‐violating experiences (Craske et al. 2014; Foa and Kozak 1986). Predirecting attention may therefore have attenuated emotional variability and reduced learning potential for state body dissatisfaction. However, as AB was not significantly modified, this interpretation remains tentative.

In contrast, trait‐level outcomes (trait body dissatisfaction, drive for thinness, trait fear of gaining weight and body appreciation) did not significantly change. These constructs reflect stable cognitive–motivational features closely tied to identity, chronicity and entrenched maladaptive beliefs (Williamson et al. 2004; Fairburn et al. 2003). This pattern, observed in the present study, is consistent with exposure models, which suggest that situational fear responses tend to change more rapidly than more stable cognitive constructs such as body dissatisfaction or drive for thinness. Durable fear reduction typically requires repeated and varied exposure experiences across sufficiently dense trials, allowing for progressive reductions in fear responses through learning processes (Craske et al. 2014; Craske et al. 2022). Consistent with this framework, the five‐session VR‐MET protocol may have been sufficient to reduce situational anxiety and fear responses but insufficient to modify entrenched trait‐level disturbances (Bouton 2004; Young et al. 2003). Only 36.8% of participants completed the full weight‐gain hierarchy, as progression required within‐session anxiety reduction. Persistently elevated anxiety within sessions limited advancement and reduced exposure dose. Insufficient exposure intensity may also have constrained generalization of corrective learning beyond the immediate context (Craske et al. 2014; Craske et al. 2022; Young et al. 2003). Within the parameters of the present protocol, VR‐MET primarily appears to modulate state‐dependent processes rather than broader trait‐level restructuring.

Reductions in overall state anxiety were not accompanied by corresponding changes in body‐part–specific anxiety, suggesting modulation of generalized affective responses while region‐specific concerns remained stable. No differential BMI effects were observed, likely reflecting the short duration of the adjunctive intervention within comprehensive multidisciplinary care. Improvements in body distortion across groups may represent non‐specific treatment effects.

Contrary to hypotheses, ABMT did not significantly modify AB indices nor enhance outcomes beyond those achieved with VR‐MET. Although ABMT did not produce statistically significant changes in AB indices, a numerical reduction in the NF toward weight‐related areas was observed in the TAU + ABMT + VR‐MET group. This trend was in the expected direction, suggesting that ABMT may have exerted some influence on attentional allocation, even though the effect did not reach statistical significance. This result likely reflects characteristics of the sample rather than a lack of efficacy of ABMT per se, as previous studies have reported reductions in AB in both clinical and non‐clinical populations (Meschberger‐Annweiler et al. 2023; Miquel‐Nabau et al. 2023; Ascione et al. 2023). In the present study, baseline AB toward weight‐related body areas was relatively low, limiting the potential for measurable change and suggesting a floor effect. Importantly, participants were not selected based on the magnitude of their AB. Stratified intervention models propose that AB modification is most effective when applied to individuals who show a substantial bias at baseline, as attentional retraining may yield greater benefits in those with pronounced maladaptive attentional patterns (Trusheim et al. 2007). Although the use of eye‐tracking allowed objective assessment of attentional allocation (Lutz et al. 2017), integrating ABMT with VR‐MET did not translate into additional clinical benefit in this unselected sample. Moreover, the absence of clear AB modification limits the interpretation of any additive clinical benefit of ABMT.

Notably, TAU‐only participants showed increased NF toward weight‐related areas over time, whereas CFT remained stable and evenly distributed. NF reflects rapid, vigilance‐based orienting (bottom‐up), while CFT captures sustained, voluntary engagement (top‐down) (Poole et al. 2004; Yarbus 1967; Fisher et al. 2017; Goldberg and Kotval 1999). This dissociation may indicate a maladaptive hypervigilant attentional style, with automatic scanning of feared body regions without deeper processing. Such a pattern may amplify the salience of negatively appraised areas and reinforce dysfunctional body‐image schemas, consistent with models linking AB to body dissatisfaction (Williamson et al. 2004; Rodgers and DuBois 2016; Tuschen‐Caffier et al. 2015; Smith and Rieger 2006; Ascione et al. 2023; Smeets et al. 2011; Smith and Rieger 2009). Accordingly, NF may represent a particularly sensitive marker of early, involuntary AB, even when sustained attention appears relatively balanced. CFT stability may partly reflect task instructions requiring attention to the entire mirrored image, constraining top‐down allocation and masking bottom‐up vigilance effects. The observed increase in NF in TAU participants may reflect heightened attentional vigilance toward body‐related threat cues during treatment, potentially driven by maladaptive body‐related schemas and the increased salience of weight‐ and shape‐related concerns during weight restoration. This pattern can also be interpreted within the framework of objectification theory (Fredrickson and Roberts 1997), which posits that individuals, particularly women, may internalize an observer's perspective on their own bodies, leading to increased body surveillance and frequent visual monitoring of evaluatively salient body parts. In contrast, attentional indices remained stable in both VR‐based conditions, suggesting that immersive exposure may help stabilize attentional vigilance and prevent maladaptive scanning patterns, rather than the observed stability being solely a consequence of low baseline AB.

Several limitations warrant consideration. The partially randomized design introduces potential residual confounding despite statistical control of cohort differences. The modest sample size, informed by effect estimates from a small non‐clinical study, likely allowed detection of large effects but limited power for medium group × time interactions, increasing the risk of Type II error. Limited exposure dosage and incomplete hierarchy progression may have further reduced sensitivity for trait‐level change. Low baseline AB may have constrained ABMT effects. Absence of follow‐up assessments precludes conclusions regarding durability, and potential moderators (e.g., illness duration) were not examined. Restriction to adolescent females enhances internal validity but limits generalizability to males, adults and chronic presentations (Meneguzzo and Todisco 2024).

Future research should employ fully randomized designs, larger samples, follow‐up assessments and stratification based on baseline AB to clarify whether ABMT provides incremental benefit in subgroups with marked AB. Increasing session frequency and total exposure dose (e.g., 10–12 sessions delivered multiple times per week) may be necessary to determine whether more intensive VR‐MET can influence trait‐level body image disturbances (Craske et al. 2022; Pelzer et al. 2023).

In conclusion, within the short‐term window examined, adjunctive VR‐MET was associated with reductions in state‐dependent processes, particularly situational anxiety, fear of gaining weight and state body dissatisfaction. Trait‐like symptoms were more resistant to change under current dosing parameters. The absence of additional ABMT benefit likely reflects low baseline AB and lack of stratification, underscoring the need to identify conditions under which attentional retraining may potentiate VR‐based exposure.

## Author Contributions

Conceptualization, M.A., M.C.‐R., F.‐A.M.‐A., E.S.‐T., H.M.‐N., M.T.M.‐M., B.P.‐G., M.F.‐G., J.G.‐M.; methodology, M.A., M.C.‐R., F.‐A.M.‐A., E.S.‐T., H.M.‐N., M.T.M.‐M., B.P.‐G., M.F.‐G., J.G.‐M.; software, M.A., F.‐A.M.‐A., M.T.M.‐M., B.P.‐G., M.M.‐S., M.F.‐G., J.G.‐M.; validation, E.S.‐T., B.P.‐G., M.F.‐G., J.G.‐M.; formal analysis, M.A., B.P.‐G.; investigation, M.A., M.C.‐R., F.‐A.M.‐A., A.B.‐M., F.G.‐Á.; resources, M.F.‐G., J.G; data curation, M.A.; writing – original draft preparation, M.A.; writing – review and editing, M.A., M.C.‐R., F.‐A.M.‐A., E.S.‐T., H.M.‐N., M.T.M.‐M., B.P.‐G., M.F.‐G., J.G.‐M.; visualization, M.A.; supervision, J.G.‐M.; project administration, J.G.‐M.; funding acquisition, J.G.‐M.

## Funding

This work was supported by the Ministerio de Ciencia e Innovación (PID2019‐108657RB‐I00) and the Fundació la Marató de TV3 (202217‐10).

## Ethics Statement

The study was conducted according to the guidelines of the Declaration of Helsinki and approved by the Institutional Review Board of the University of Barcelona and by the Clinical Research Ethics Committee of the Hospital Sant Joan de Déu.

## Conflicts of Interest

The authors declare no conflicts of interest.

## Supporting information


**Table S1:** Baseline demographic and clinical characteristics of the new and old cohorts.

## Data Availability

The data that support the findings of this study are available from the corresponding author upon reasonable request.
