# Supplementary material for: Virtual Reality Body Exposure and Attentional Bias Modification in the Treatment of Adolescents With Anorexia Nervosa
Source: Clin Psychol Psychother. 2026 Apr 14;33(2):e70273. doi: 10.1002/cpp.70273 (PMC13078948; doi:10.1002/cpp.70273)
Supplement: Supplementary file 1 — Table S1: Baseline demographic and clinical characteristics of the new and old cohorts. [file CPP-33-e70273-s001.docx]

**SUPPLEMENTARY MATERIAL**

**Supplementary Table S1**. Baseline demographic and clinical characteristics of the new and old cohorts.

| **Variable** | **New cohort**  **(n = 54)** | **Old cohort**  **(n = 21)** | **Statistic** | **p** |
| --- | --- | --- | --- | --- |
| **Anthropometrics, M (SD)** | | |  |  |
| Age, years, | 15.15 (1.56) | 15.19 (1.12) | *t*(73) = −0.11 | .910 |
| BMI ^a^, kg/m^2^ | 16.70 (1.39) | 17.58 (0.78) | *t*(73) = −2.74 | **.008** |
| **Main Diagnosis, n (%)** | | | *χ²*(1) = 0.04 | .834 |
| AN-R type ^b^ | 52 (96.3) | 20 (95.2) |  |  |
| AN-P type ^c^ | 2 (3.7) | 1 (4.8) |  |  |
| **Number of Comorbid Diagnoses, n (%)** | | | *χ²*(2) = 0.11 | .949 |
| 0 | 35 (64.8) | 13 (61.9) |  |  |
| 1 | 16 (29.6) | 7 (33.3) |  |  |
| 2 | 3 (5.6) | 1 (4.8) |  |  |
| **Any psychotropic medications** ^d^**, n (%)** | | | *χ²*(1) = 19.78 | **< .001** |
| Yes | 49 (90.7) | 9 (42.9) |  |  |
| No | 5 (9.3) | 12 (57.1) |  |  |
| **Treatment Program, n (%)** | | | *χ²*(2) = 21.71 | **< .001** |
| Day-patient (11 h/day) | 22 (40.7) | 21 (100) |  |  |
| Day-patient (6 h/day) | 12 (22.2) | 0 (0) |  |  |
| Home-treatment | 20 (37.0) | 0 (0) |  |  |
| **Eating disorder psychopathology (EDI** ^e^**), M (SD)** | | |  |  |
| Body Dissatisfaction | 28.31 (6.78) | 26.00 (4.80) | *t*(73) = 1.43 | .157 |
| Drive for Thinness | 22.61 (6.75) | 21.76 (4.05) | *t*(73) = 0.54 | .592 |
| Fear of Weight Gain | 3.48 (1.15) | 3.76 (0.89) | *t*(73) = −1.01 | .316 |

Note. Values are means (M) and standard deviations (SD) for continuous variables and counts and percentages (n [%]) for categorical variables. ^a^ BMI = body mass index; ^b^ AN-R = anorexia nervosa restrictive subtype; ^c^ AN-P = anorexia nervosa purgative subtype; ^d^ Any psychotropic medication indicates treatment with at least one psychotropic drug at baseline; ^e^ EDI = Eating Disorder Inventory.
